# Supplementary material for: Utilisation of Skilled Birth Attendant in Low- and Middle-Income Countries: Trajectories and Key Sociodemographic Factors
Source: Int J Environ Res Public Health. 2021 Oct 13;18(20):10722. doi: 10.3390/ijerph182010722 (PMC8535845; doi:10.3390/ijerph182010722)
Supplement: Supplementary file 1 [file ijerph-18-10722-s001.zip › ijerph-1371000-supplementary.pdf]

Table S1 : Variation inflation factors (VIFs) for the models in the study, as reported in Table 3 of the

	Indonesia		Nigeria	
Characteristic	VIF	Df	VIF	Df
Year of survey	1.091	3	1.078	3
Age	1.232	1	1.197	1
Father attained secondary education or higher	1.516	1	1.674	1
Autonomy	1.008	1	1.248	1
Secondary or higher education	1.615	1	2.975	1
Media exposure at least once a week	1.165	1	1.318	1
Birth interval	1.036	1	1.011	1
First or second birth order	1.236	1	1.198	1
At least one Antenatal appointment	1.128	1	1.441	1
Wealth Quintile	1.498	1	1.927	1
Residence	1.285	1	1.451	1
Literacy	1.174	1	3.016	1
Distance to HCF	1.080	1	1.128	1
Geographic SBA Distribution	1.035	3	1.717	3

Figure S1: Trend of Maternal Mortality Rates in Indonesia and Nigeria (2002 – 2018).

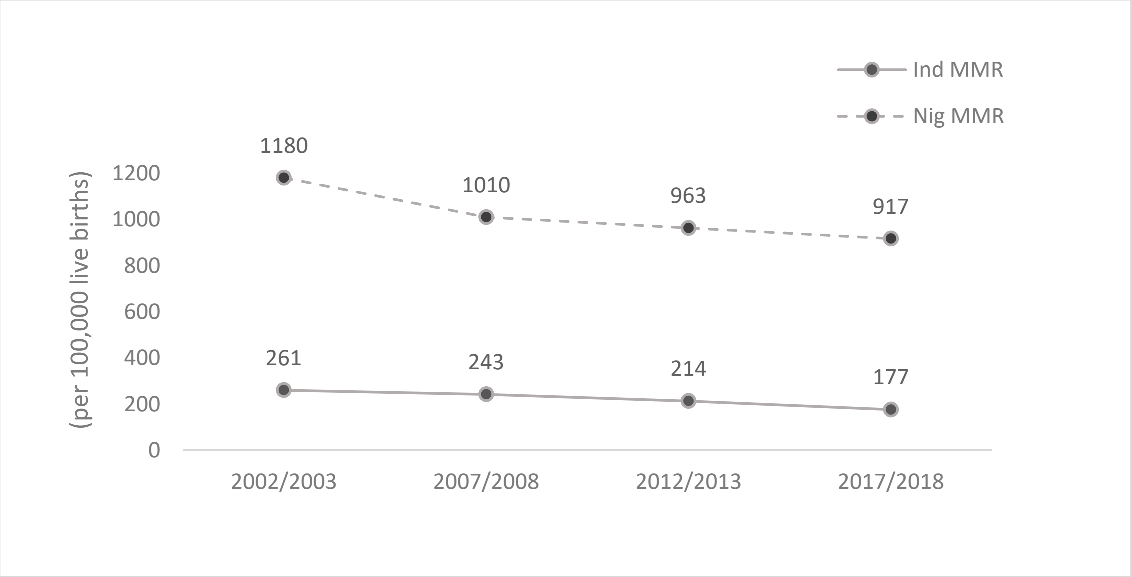

Source:

https://data.worldbank.org/indicator/SH.STA.MMRT?end=2017&locations=ID&start=2000&view=chart

https://data.worldbank.org/indicator/SH.STA.MMRT?end=2017&locations=NG&start=2000&view=chart

Table S2: Results of logistic regression models for SBA: predictors of SBA utilisation for both Indonesia and Nigeria during first and last period

Characteristic	Indonesia 2002/2003 (N=12760))		Indonesia 2017/2018 (N=15021)		Nigeria 2002/2003 (N=3911))		Nigeria 2017/2018 (N=21911)	
	Unadjusted OR (95% CI)	Adjusted OR (95% CI)	Unadjusted OR (95% CI)	Adjusted OR (95% CI)	Unadjusted OR (95% CI)	Adjusted OR (95% CI)	Unadjusted OR (95% CI)	Adjusted OR (95% CI)
**Sociodemographic Factors**								
Age (30-49 vs 15-29)	0.99 (0.92-1.07)	1.53 (1.34-1.74)***	1.31 (1.24-1.38)	1.65 (1.35-2.01)***	1.07 (0.94-1.22)	1.11 (0.87-1.42)	1.31 (1.24-1.38)***	1.26 (1.14-1.39)***
Father attained secondary education or higher (yes vs no)	5.53 (5.09-6.01)***	2.17 (1.9-2.47)***	7.22 (6.78-7.68)***	1.94 (1.62-2.33)***	5.11 (4.42-5.9)***	1.6 (1.25-2.05)***	7.22 (6.78-7.68)***	1.4 (1.26-1.56)***
**Empowerment**								
Autonomy (yes vs no)	1.57 (1.45-1.7)***	1.2 (1.06-1.35)***	4.04 (3.8-4.3)***	1.21 (1.02-1.44)*	3.12 (2.59-3.75)***	1.79 (1.32-2.42)***	4.04 (3.8-4.3)***	1.12 (1.01-1.25)*
Secondary or higher education (yes vs no)	6.16 (5.64-6.73)***	2.19 (1.9-2.53)**	10.88 (10.2-11.6)***	2.3 (1.91-2.78)***	9.81 (8.3-11.6)***	2.74 (1.94-3.87)***	10.88 (10.2-11.6)***	1.71 (1.5-1.95)***
Media exposure at least once a week (yes vs no)	3.01 (2.74-3.3)***	1.08 (0.93-1.24)	4.37 (4.13-4.63)***	1.17 (0.96-1.44)	2.57 (2.23-2.97)***	0.94 (0.73-1.22)	4.37 (4.13-4.63)***	1.27 (1.15-1.4)***
**Family Planning**								
Birth interval less than 24 months (yes vs no)	1.06 (0.92-1.22)	1 (0.83-1.2)	0.94 (0.87-1.01)***	0.69 (0.53-0.91)**	1.14 (0.95-1.37)	1.34 (1.01-1.77)*	0.94 (0.87-1.01)	0.91 (0.82-1.02)
First or second birth order (yes vs no)	1.99 (1.84-2.15)***	1.4 (1.23-1.59)***	1.84 (1.74-1.95)***	1.39 (1.15-1.67)***	1.62 (1.42-1.86)***	1.31 (0.96-1.79)	1.84 (1.74-1.95)***	1.11 (0.99-1.26)
At least one Antenatal appointment (yes vs no)	13.05 (11.04-15.42)***	7.17 (5.75-8.94)***	19.12 (17.49-20.92)***	8.78 (6.37-12.12)***	29.28 (23.06-37.18)***	12.06 (8.75-16.61)***	19.12 (17.49-20.92)***	11.1 (9.81-12.57)***
**Economic Accessibility**								
Wealth Quintile (Upper middle vs Poor)	4.58 (4.23-4.96)***	2.07 (1.81-2.35)***	9.06 (8.5-9.66)***	2.57 (2.09-3.16)***	5.62 (4.82-6.54)***	1.64 (1.25-2.15)***	9.06 (8.5-9.66)***	1.77 (1.59-1.98)***
**Community Impacting Factors**								
Residence (Urban vs Rural)	2.95 (2.73-3.2)***	1.29 (1.14-1.47)***	5.36 (5.05-5.69)***	1.76 (1.45-2.15)***	4.2 (3.63-4.86)***	1.42 (1.1-1.82)**	5.36 (5.05-5.69)***	1.3 (1.18-1.44)***
Literacy (Can read part or whole sentences vs not)	4.23 (3.69-4.84)***	1.3 (1.09-1.56)***	9.69 (9.1-10.31)***	1.29 (0.95-1.75)	8.9 (7.66-10.34)***	1.21 (0.89-1.63)***	9.69 (9.1-10.31)***	1.54 (1.35-1.75)***
Distance to HCF (Big problem vs small or no problem)	0.31 (0.28-0.34)***	0.48 (0.41-0.56)	0.51 (0.48-0.55)***	0.65 (0.53-0.8)***	0.29 (0.24-0.35)***	0.52 (0.39-0.69)***	0.51 (0.48-0.55)***	0.85 (0.76-0.95)**
Geographic SBA Distribution (Ref: Quartile 1)								
Regional Quartile 2	0.88 (0.78-0.99)*	0.89 (0.75-1.05)	2.07 (1.91-2.24)***	1.54 (1.26-1.88)***	3.34 (2.7-4.12)***	1.68 (1.26-2.25)***	2.07 (1.91-2.24)***	1.39 (1.24-1.56)***
Regional Quartile 3	2.09 (1.85-2.35)***	2.12 (1.79-2.53)***	8.87 (8.11-9.7)***	4.92 (3.81-6.33)***	11.01 (9-13.49)***	6.14 (4.56-8.26)***	8.87 (8.11-9.7)***	4.96 (4.36-5.65)***
Regional Quartile 4	3.99 (3.43-4.64)***	4.12 (3.32-5.12)***	30.88 (27.85-34.24)***	3.3 (2.48-4.38)***	57.62 (42.22-78.63)***	8.01 (5.66-11.34)***	30.88 (27.85-34.24)***	11.04 (9.41-12.95)***

Notes: * = Significant at 0.05; ** = Significant at 0.01, *** = Significant at 0.001.

Table S3: SBA utilisation by care provider sub-categories for Indonesia and Nigeria (2002-2018)

	Indonesia				Nigeria			
	2002	2007	2012	2017	2003	2008	2013	2018
Obstetrician / Gynecologist	10.4%	13.1%	20.1%	28.4%	-	-	-	-
General practitioner /Doctor	1.0%	1.3%	1.2%	1.6%	7.8%	8.3%	10.5%	8.9%
Nurse	43.5%	11.9%	11.8%	22.4%	35.3%	27.9%	33.0%	37.5%
Midwife		45.7%	48.5%	59.7%				
Aux midwife	-	-	-	-	5.9%	-	4.1%	3.1%
Village midwife	20.8%	18.0%	17.7%	14.1%	-	-	-	-
Traditional birth attendant	38.2%	35.6%	24.3%	14.0%	17.40%	22.90%	22.70%	17.90%
CS Health professional	-	-	-	-	1.3%	-	-	-
Community extension health worker	-	-	-	-	3.50%	-	3.50%	-
